# Statistical Complexity of the Coriolis Antipairing Effect

**DOI:** 10.3390/e21060558

**Published:** 2019-06-03

**Authors:** Flavia Pennini, Angelo Plastino

**Affiliations:** 1 Departamento de Física, Universidad Católica del Norte, Av. Angamos 0610, Antofagasta 1240000, Chile; 2 Departamento de Física, Facultad de Ciencias Exactas y Naturales, Universidad Nacional de La Pampa, CONICET, Av. Peru 151, Santa Rosa, La Pampa 6300, Argentina; 3 Instituto de Física La Plata–CCT-CONICET, Universidad Nacional de La Plata, C.C. 727, La Plata 1900, Argentina; 4 Social Thermodynamics Applied Research-École polytechnique fédérale de Lausanne—SThAR-EPFL, 1015 Lausanne, Switzerland

**Keywords:** fermions, statistical complexity, exactly solvable many-body model, nuclear superconductivity

## Abstract

Using the entropic quantifier called statistical complexity, we investigate the interplay between (1) pairing interactions between fermions, can be viewed as analogous with superconductivity based on Cooper pairs; (2) rotations of the system as a whole around an axis; and (3) thermal excitations. Two different ordering processes are at work: alignment and pairing of two fermions to total spin zero. They compete among themselves and with thermal disorder. A complex physics ensues as a consequence. The existence of novel phenomena is revealed by the behavior of the statistical complexity. In particular, it is seen how order can arise out of disorder in originating high-temperature superconductivity.

## 1. Introduction

Most significant properties of quantum systems are evaluated at zero-temperature *T*, superconductivity excepted [1,2,3]. It is known that many of them keep maintaining them in place at low enough *T*, where statistical considerations become inevitable. Assume that the important dynamic features of a quantum many body system (QMB) are known at zero *T*. What statistical quantifiers suffice to appropriately describe them at finite temperatures?

We wish in this effort to investigate the interplay between (1) pairing interactions between fermions, responsible for superconductivity based on nuclear Cooper pairs; (2) rotations of a QMB system as a whole around an axis; and (3) thermal excitations. Such an interplay gives rise to the so-called Coriolis antipairing effect, to be discussed at length below. A first effort in this sense is that of reference [4]. We will here *add a powerful tool* that enables one to access new and wonderful vistas: the relatively recent concept of statistical complexity *C* which will be seen to originate new and revealing insights.

The standard formulation today of the statistical complexity notion was originally advanced by López-Ruiz-Mancini-Calvet (LMC) and has received a lot of attention in its 25 years of existence [5,6,7,8]. The consensus is that the ensuing *C* is a statistical indicator that can yield different and perhaps deeper insights than purely dynamic ones. Certainly, *this C* is a quantifier that complements entropy *S* in the sense that it grasps correlation structures in the manner that *S* does it with disorder.

Reference [5] was regarded by many researchers as a great leap forward. Its authors most interesting move was perhaps to appeal to a sort of “distance” from the actual to the maximum entropy instance, a distance that was called disequilibrium *D* [6]. In these two last references the distance is assessed in a probability space, being the one that separates the prevailing probability distribution from the uniform one. The quantifier *D* is non-null only in the cases in which “privileged” states exist amongst the available ones, so that *D* becomes maximal for fully ordered systems and is null in totally random environments. Indeed, for the entropy *S* things are exactly reversed. The standard form for a measure of statistical complexity *C* became then

(1) C=DS,

a functional of the probability distribution [5]. If our system contains (i) a finite number of particles *N* and (ii) a number of eigenstates of the pertinent Hamiltonian H (in the model to be discussed here *N* is finite), then one writes

(2) $$D=\sum_{i=1}^{N}\;{\left(p_{i}-\frac{1}{N}\right)}^{2},$$

where $p_{1},p_{2},\ldots ,p_{N}$ are the individual normalized probabilities ($\sum_{i=1}^{N}\;p_{i}=1$) [5], becoming a maximum for a fully ordered state and zero for equiprobable ones. By *S* we understand here the Shannon–Boltzmann entropy (or information), given by [9,10,11]

(3) $$S=-\sum_{i=1}^{N}\;p_{i}\;\ln p_{i}.$$

As already stated above, LMC’s notions received a lot of attention (see References [12,13,14,15,16,17,18], as a small sample). They have been used in different scientific scenarios for the microcanonical, canonical, and grand canonical ensembles.

### 1.1. Motivation and Goal

In the analysis of nuclear structure effects at high excitation energy and high angular momentum, exemplified, for instance, by the case of compound nuclei formed during a heavy-ions collision, the inclusion of rotational and thermal degrees of freedom has conclusively shown to be a useful theoretical approach [19,20]. If one places these problems on an equal footing with the ones posed by the conventional gamma-spectroscopy and particle spectroscopy at low energy, the valuable available literature does exhibit the usefulness of simple, exactly soluble models, where only the main facets and properties of extended and more complex calculations are emphasized [4].

We thus focus attention upon the problem of investigating an exactly soluble model in which the rotational and thermal degrees of freedom are simultaneously treated, as well as their mutual interplay, both quantitatively and qualitatively. With this goal in mind, we address the simultaneous analysis of the pairing force (superconductivity) and of the cranking (rotational) interaction at finite temperature by considering the model advanced in Reference [4].

We will deal with an exactly solvable SU(2) × SU(2) model that exhibits both superconducting and rotational features. These two distinctive attributes strongly interact among themselves. Our goal is that of investigating how the statistical complexity responds to this competition. It will be seen that some new physical properties will be discerned.

### 1.2. Paper’s Structure

The paper is organized as follows. Section 2 describes our exactly solvable many fermion system at zero temperature. Section 3 discusses the finite temperature case. Section 4 adapts the statistical complexity formalism to the specificity of our many-body model. Section 5 looks at the finite temperature effect, together with the associated results that constitute the goal of this effort. Finally, we draw some conclusions in Section 6.

## 2. The Model

We deal with a two-level model. Each level is degenerate and can accommodate N=2Ω fermions. The two levels are separated by an energy gap ϵ, that we take equal to unity (in arbitrary units). Since we are dealing with an SU(2) × SU(2) fermion-model, we have [4] four quantum numbers specifying each state of the fermion-system, that one calls $J,M,Q,Q_{o}$, i.e., our states are of the form $|J,Q,M,Q_{o}\rangle$. In our case we have always $Q_{o}=0$ [4]. The model’s Hamiltonian $\hat{H}$ has three terms: a single particle one (*sp*) (*sp* states are denoted by ν and have *sp*-energy $e_{\nu }$), a pairing one, and a rotational one [4]

(4) $$\hat{H}={\hat{H}}_{sp}+{\hat{H}}_{rot}+{\hat{H}}_{pairing},$$

with (in arbitrary energy units)

(5) $${\hat{H}}_{sp}=\sum_{\nu }e_{\nu }\;{\hat{a}}_{\nu }^{+}{\hat{a}}_{\nu },$$

(6) $${\hat{H}}_{rot}=-\omega \sum_{\nu ,\;\nu^{\prime }}\langle \nu |{\hat{J}}_{x}|\nu^{\prime }\rangle \;{\hat{a}}_{\nu }^{+}{\hat{a}}_{\nu^{\prime }},$$

with ω the rotational frequency and ${\hat{J}}_{x}$ the *x*-component of an angular momentum $\hat{J}$ (cranking). If $\bar{\nu }$ stands for the time reversed of the state ν and *G* (energy units) for the pairing coupling constant we have [4],

(7) $${\hat{H}}_{pairing}=-\frac{G}{2}\sum_{\nu ,\;\nu^{\prime }\;>0}{\hat{a}}_{\nu }^{+}{\hat{a}}_{\bar{\nu }}^{+}{\hat{a}}_{\bar{\nu^{\prime }}}{\hat{a}}_{\nu^{\prime }}.$$

The $\hat{a}$—representation is not the most convenient one to work with. After a rather complicated procedure, fully explained in Reference [4], one passes to a much more advantageous operators ${\hat{b}}_{r,\;K}^{+}$ and ${\hat{b}}_{r,\;K}$, with r=1,2. People usually understand that this kind of operators create or destroy **quasi-particles**. The most celebrated case is that of operators that result from a Bogoliubov-Valatin transformation of this sort, that results in the celebrated BCS description of super-conductivity [21]. We use sub-indexes *K* and (time-reversed ones) $\bar{K}$. In Reference [4] they call $\hat{b}$ the operators involving $\bar{K}$. In terms of these new *b*’s the quasi-particle Hamiltonian becomes

(8) $$\hat{H}\left(b\right)=E_{o}{\hat{J}}_{z}\left(b\right)-\frac{G}{2}{\hat{Q}}_{+}\left(b\right){\hat{Q}}_{-}\left(b\right),$$

with

(9) $$E_{o}=\sqrt{\omega^{2}+\epsilon^{2}},$$

(10) $${\hat{J}}_{z}\left(b\right)=\sum_{r,\;K}\;{\left(-1\right)}^{r}\;\left[{\hat{b}}_{r,\;K}^{+}{\hat{b}}_{r,\;K}+{\hat{b}}_{r,\;K}^{+}{\hat{b}}_{r,\;K}\right],$$

(11) $${\hat{Q}}_{+}\left(b\right)=\sum_{K}\;\left[{\hat{b}}_{2,\;K}^{+}{\hat{b}}_{1\;K}^{+}+{\hat{b}}_{2,\;K}^{+}{\hat{b}}_{1,\;K}^{+}\right],$$

(12) $${\hat{Q}}_{-}\left(b\right)={\hat{Q}}_{+}^{+}\left(b\right).$$

Note that the *Q* operators commute with the $\hat{J}$ ones [4]. With this new quasi-particle Hamiltonian the problem is easily solved exactly, at T=0, as shown in Reference [22]. As explained in References [4,22], an interesting manner of labeling the states of our system appeals to a quantum number, called the quasi-spin seniority *s*, that represents the number of unpaired fermions (to spin-projection M=0). The unperturbed ground state has s=2Ω. The fully paired, superconducting state has s=0. Additionally, one can see that

(13) J=s/2,

and

(14) J+Q=Ω,

which implies that one does not need to sum over the quantum number *Q*. Remember also that $Q_{o}=0$, fixed [4].

The exact energies acquire the form [4]

(15) $$E_{J,M}=-E_{o}M-\frac{G}{2}\;\left(\Omega -J\right)\left(\Omega -J+1\right).$$

Full superconductivity suddenly arises whenever, growing from zero, *G* reaches the critical value

(16) $$G_{crit}=\frac{2E_{o}}{\Omega +1},$$

so that, as ω grows, it takes more paring strength to reach the superconductor’s state. The system becomes then fully ordered (coherent nuclear Cooper pairs). Another critical transition takes place whenever ω grows from zero and the system then begins to rotate. When ω reaches the critical value [4]

(17) $$\omega_{crit}=\sqrt{\frac{G^{2}}{4}{\left(\Omega +1\right)}^{2}-\epsilon^{2}},$$

the system suddenly jumps from s=0 to s=Ω, destroying superconductivity. This vanishing is the essence of the Coriolis antipairing effect. Below, we will take G=2, Ω=4, and ϵ=1, so that $\omega_{crit}=4.89$.

At such value, the system goes over to another kind of order, alignment. We have a sudden transition from one kind of order to another type of it. At play here we appreciate the competition of two different kinds of order, i.e.,

- pairing of fermions to M=0 and
- alignment along the *x*-axis.

Note that the competition between the two modes of order is asymmetric in the sense that rotation can destroy superconductivity in sudden fashion but not vice versa.

## 3. The Finite Temperature Case

Since we deal with a fixed number of particles N=2Ω, the canonical partition function is to be employed here [4]. One sums over just *J* and *M*, as explained above. Then, we have

(18) $$Z=\mathrm{Tr}\;e^{-\beta \hat{H}}=\sum_{J=0}^{\Omega }\sum_{M=-J}^{J}\;\exp\left(-\beta E_{J,M}\right),$$

where we use the energies (15) and set $\beta =1/k_{B}T$, where *T* is the temperature (expressed in units of energy). The equilibrium entropy is [9]

(19) S=lnZ+βU,

with *U* the mean energy

(20) $$U=-\frac{\partial \ln Z}{\partial \beta }.$$

An important quantity is the mean quasi-spin seniority $\bar{s}$ which on account of Equation (13) becomes [4]

(21) $$\bar{s}=\frac{1}{Z}\sum_{J=0}^{\Omega }\sum_{M=-J}^{J}\;2J\;\exp\left(-\beta E_{J,M}\right).$$

All the relevant properties of the system can be evaluated as a function of both β, ω, and Ω, in terms of the exact solutions, within the present SU(2) × SU(2) scheme.

## 4. Disequilibrium and Statistical Complexity at Finite Temperature

Our goal here is to obtain the disequilibrium and the LMC-statistical complexity for the model explained in the previous section.

For this purpose we remember that the canonical probability distribution alludes to probabilities $P_{J,M}$ of the form

(22) $$P_{J,M}=\frac{\exp\left(-\beta E_{J,M}\right)}{Z},$$

where $E_{J,M}$ is the energy given by Equation (15). Thus, the corresponding entropy *S* is also rewritten in terms of the probabilities $P_{J,M}$ as

(23) $$S=-\sum_{J=0}^{\Omega }\sum_{M=-J}^{J}\;P_{J,M}\ln P_{J,M}.$$

Moreover, according to Equation (2), we have for the disequilibrium *D* the following expression

(24) $$D=\sum_{J=0}^{\Omega }\sum_{M=-J}^{J}\;{\left(P_{J,M}-\frac{1}{{\left(\Omega +1\right)}^{2}}\right)}^{2},$$

so that the statistical complexity C=DS of our model is obtained from Equation (1) with *S* and *D* given by Equations (23) and (24), respectively.

## 5. Main Results

### 5.1. Quantifiers Versus ω at Several β-Values for Fixed G=2 and Ω=4

The values for *G* and Ω are taken from Reference [4]. We start by considering *D*.

**Figure 1:**
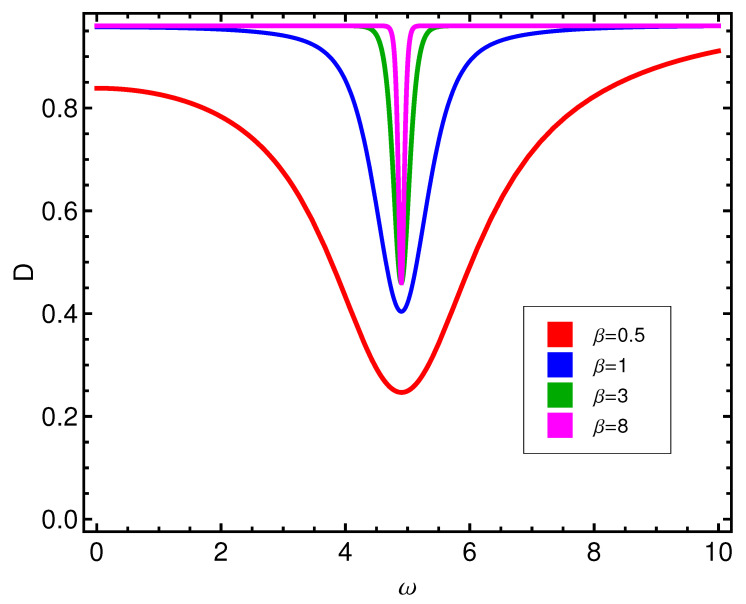
Disequilibrium *D* versus ω (in ϵ units) for different values β ($k_{B}T$ in ϵ units). One appreciates here the *D*-behavior of two competing types of order. *D* is not larger for alignment than for pairing, except at high *T*, with a profound valley at $\omega_{crit}=4.89$. It seems that high *T* disrupts pairing effects on *D* in a more severe fashion than it does for alignment-effects.

**Figure 2:**
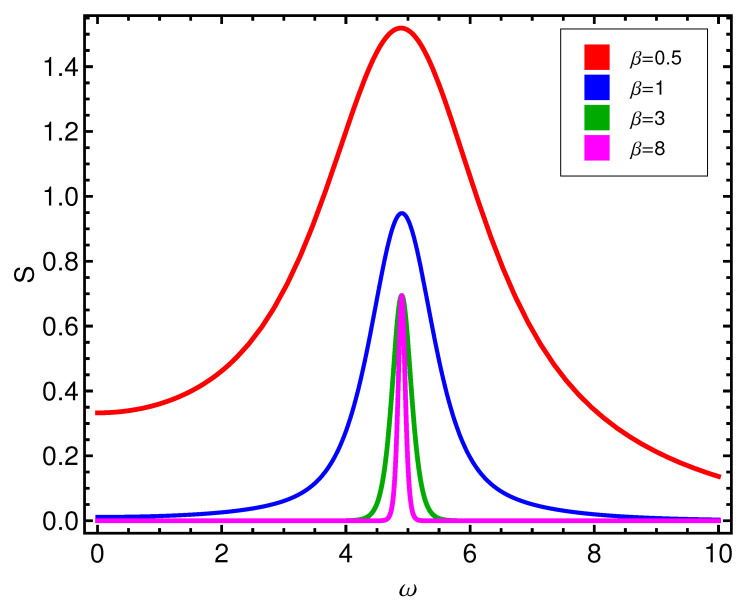
Entropy *S* versus ω (in ϵ units) for different values of β ($k_{B}T$ in ϵ units). The critical point is at $\omega_{crit}=4.89$. Ordering effects on *S* are the opposite counterpart of them on *D*.

One appreciates in Figure 1 the *D*-behavior of the two competing types of order, as reflected by the disequilibrium *D*. *D* is larger for alignment than for pairing, with a profound valley at $\omega_{crit}$. Instead, the entropy *S* has a peak at such critical $\omega_{crit}$, as illustrated by Figure 2. At the phase transition the entropy becomes maximal.

**Figure 3:**
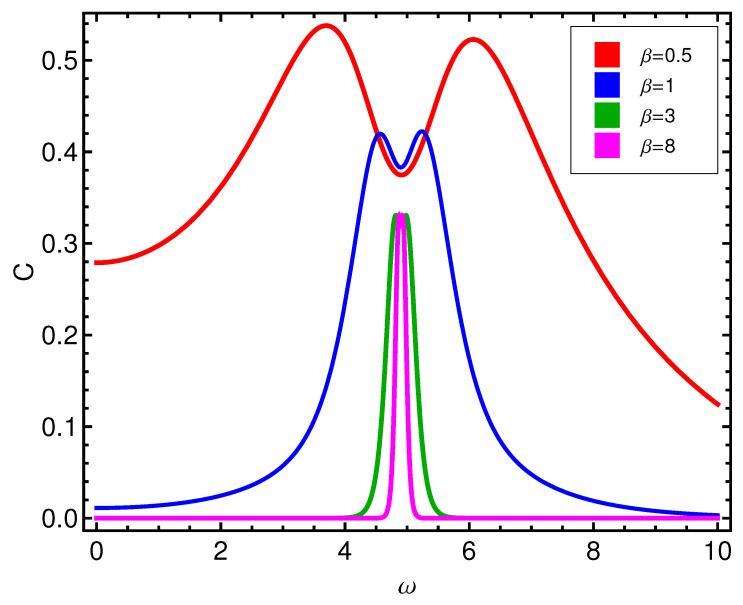
Complexity *C* versus ω (in ϵ units) for different β-values ($k_{B}T$ in ϵ units).

**Figure 4:**
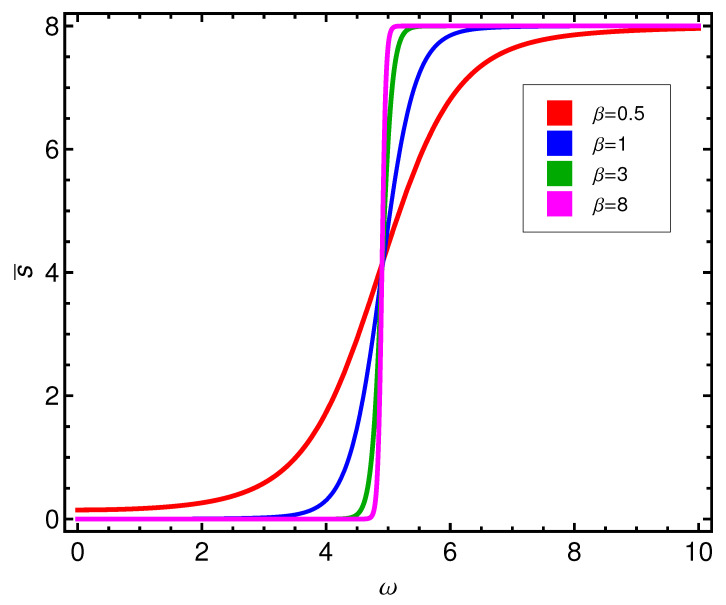
Seniority $\bar{s}$ versus ω (in ϵ units) for different values of β ($k_{B}T$ in ϵ units).

The complexity *C* displays a more involved behavior, as can be seen by glancing at Figure 3. There is a *C*-peak, but something else happens with it as *T* grows, that will be further discussed below. Pass now to the mean seniority. In inspecting $\bar{s}$ we appreciate a rather surprising result. This quantifier is a descriptor of the occupation number for quasi-particles. We see in Figure 4 that our quasi-particles display an occupation-behavior that resembles that for a Fermi ideal gas, which makes a lot of sense.

In general, the specific heat value is related to the number of degrees of freedom of the system at hand, which tells us how free the system is to transform itself in different ways (and thus how much kinetic energy can it store inside itself without breaking apart). Solids have a more fixed structure and they cannot rotate and jostle too much. Accordingly, they are not able to store much internal energy and possess a lower heat capacitance than liquids (lower $C_{V}$). *The more ordered the system is, the lower the specific heat $C_{V}$* becomes. During a phase change, the number of degrees of freedom changes, and so does the specific heat. $C_{V}$ is vanishingly small for the two types of order we are talking about here but significantly grows just before and after the phase transition, vanishing at it.

*The fact of dealing with a finite Hilbert space has some odd physical consequences that we have to point out.* We are referring specifically to the limit T→∞. For our two-level model, in this limit all micro-states |J,M〉 become equally likely (EL). Thus, a paradoxical configurational situation thereby ensues. The system attains the maximum possible degree of disorder (MPDD) and becomes thereby “frozen” in the sense that nothing else can happen to it in this EL-environment. This is reflected by the fact that, then, $C_{V}=0$, as one sees here. Implausibly enough, MPDD turns out to produce, in a sense, the same effects as total order on $C_{V}$ and we could not be too severely reproached if we call this scenario a one of order out of disorder. $C_{V}$ behaves, at maximum disorder, as it responds to maximal order.

### 5.2. Density Plot for Fixed G=2 and Ω=4

**Figure 5:**
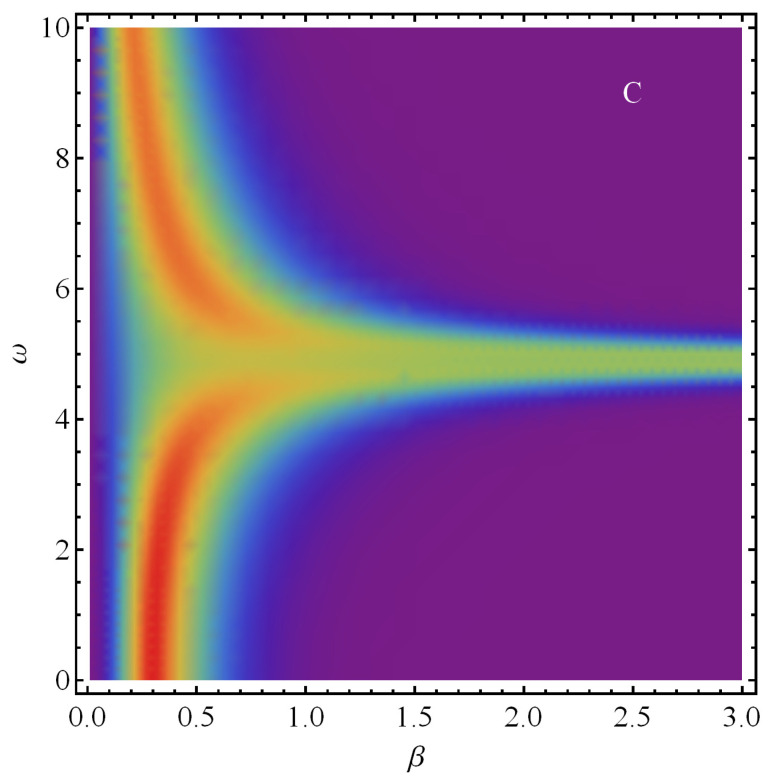
Density plot of statistical complexity *C* versus β and ω (in ϵ units).

It is the turn now of an illustrative density plot, drawn in Figure 5, representing the statistical complexity *C* versus β and ω. In this plot, the darker the color, the lower becomes the value of the third coordinate. In the graph we clearly see that, at $\omega_{crit}$, *C* is high for all β-level curves, in the range here depicted. Interestingly enough, at very high *T* (for both high and low ω) we encounter a zone of *high C*-values. This entails that at these high temperatures a process of disorder-order-disorder takes place. One may speak of an emergence of order out of disorder here. This is something we will attribute below to the high-*T* configurational effects.

### 5.3. Quantifiers Versus β at Several ω-Values for Fixed G=2 and Ω=4

**Figure 6:**
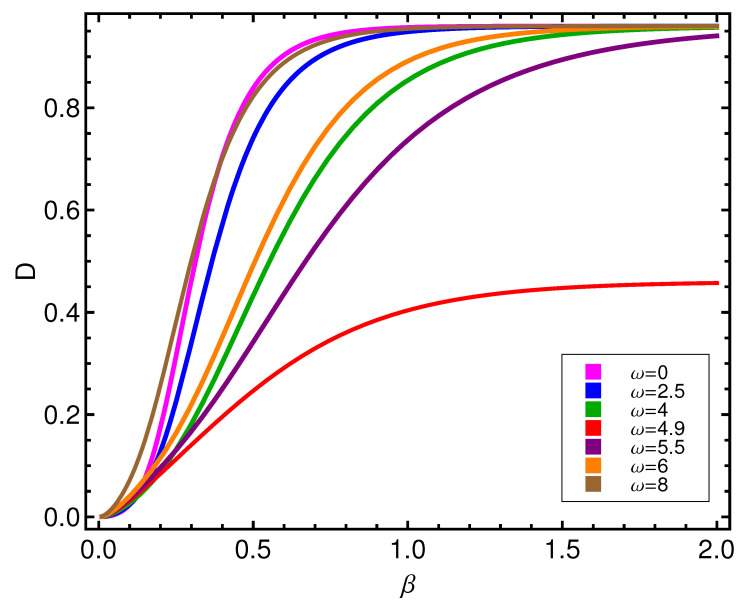
Disequilibrium *D* versus β for different values of ω (in ϵ units).

It is clear that *D* has to increase as *T* diminishes, as we appreciate in Figure 6. We only ask the reader to recognize that two kinds of curves exist, according to whether ω is greater or smaller than $\omega_{crit}=4.89$, indicative of the fact that we deal with two different modes of order.

**Figure 7:**
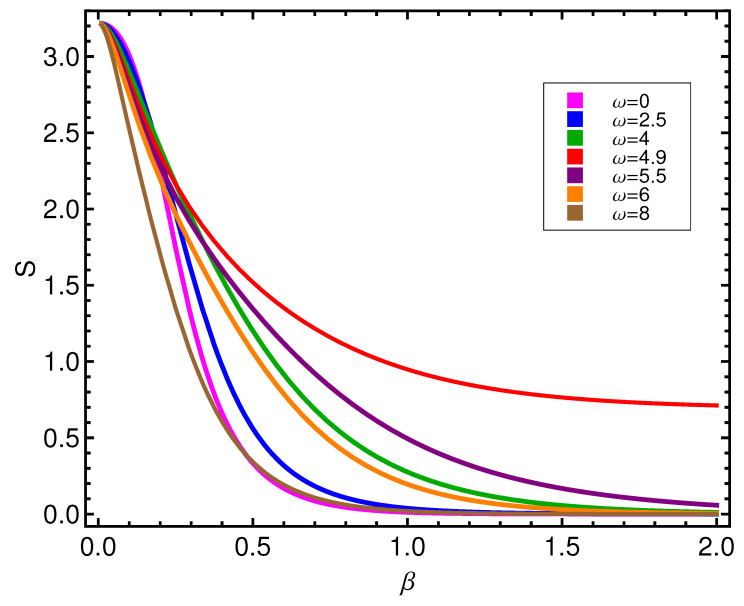
Entropy *S* versus β for different values of ω is in energy units (ϵ).

Instead, in Figure 7, we observe that the entropy singles out the curve corresponding to the transition between modes at $\omega_{crit}=4.89$ by not vanishing so quickly there, even at very low *T*. For other ω’s, the associated curves rapidly tend to vanish.

**Figure 8:**
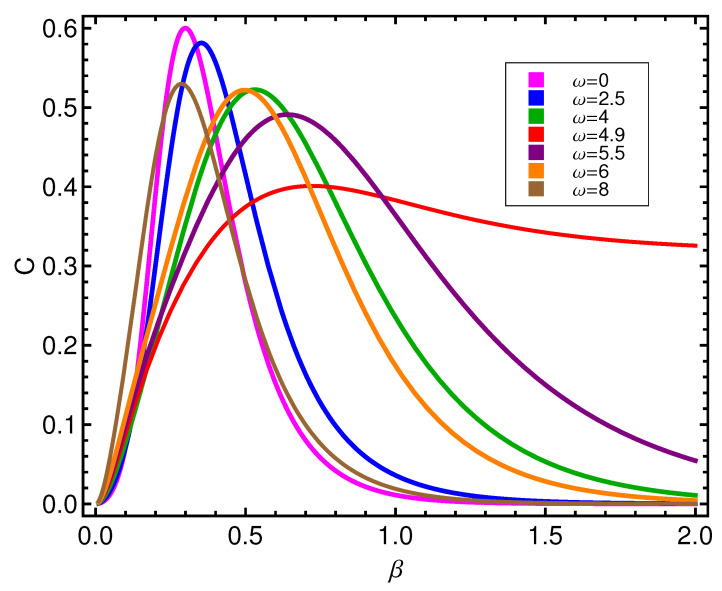
Complexity *C* versus β for different values of ω in energy units (ϵ).

The counterpart of the above scenario is displayed by Figure 8, for the statistical complexity behavior as *T* changes. The passage from a labile state of affairs to a frozen, configurational one referred to above is here reflected by complexity maxima, roughly, in the β∈0.3–0.6-region.

**Figure 9:**
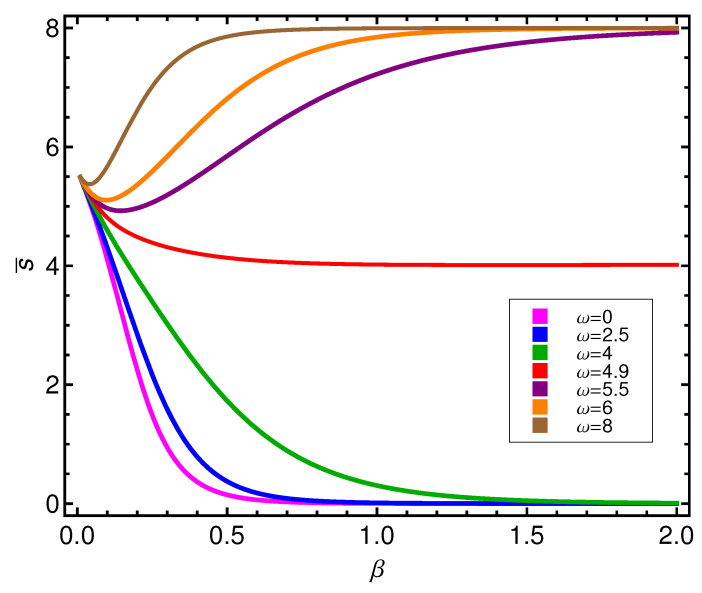
Seniority $\bar{s}$ versus β for different values of ω in energy units (ϵ).

The mean seniority *T*-behavior is illustrated by Figure 9. The quantum phase transition is clearly visible by inspection of the curve associated to $\omega_{crit}=4.9$, for which $\bar{s}=4$ most of the time, as it should. The high-*T* configurational effect appears here with full splendor, as all curves coalesce at T→∞ at a finite, common $\bar{s}$ value. Curves for $\omega >\omega_{crit}$ descend in that limit, while the ones for $\omega <\omega_{crit}$ ascend. A high-*T* partial superconductivity emerges, whose origin is clearly configurational.

### 5.4. Quantifiers Versus *G* for Several β-Values

For completeness we briefly describe what happens in this case at the superconductor transition. As for *D* vs. *G*, $G_{crit}∼2$. The superconductor transition can be clearly detected at low *T* and blurs as *T* grows. For very high *T*, equiprobability reigns.

We can also mention, for the *C*-behavior, that at high (but not *too high T*), the configurational “order from disorder” effect discussed above reappears. Of course, at T→∞, *C* tends to vanish.

## 6. Conclusions

In this work we have reaffirmed the fact that statistical complexity *C* is a powerful tool for the study of phase-transitions. Two different ones have been considered here and we have gained interesting insight from the concomitant analysis. One is the superconducting one (Equation (16)) and the other is the Coriolis antipairing process through which rotation weakens superconductivity.

A noticeable configurational effect appears at high temperatures that is newly revealed here for the present model.

The above mentioned configurational effect is the result of a finite Hilbert space’s restriction that severely limits the number of accessible micro-states. Its main consequence is the existence for this model of high temperature (partial) superconductivity (HTS).

Could the same happen in other circumstances? We tentatively suggest that composite substances displaying HTS would be highly unstable at too high energies, probably ceasing to exist then. Effectively thus, their accessible Hilbert space could become restricted.
